# Evaluating Clinical Trial Designs for Investigational Treatments of Ebola Virus Disease

**DOI:** 10.1371/journal.pmed.1001815

**Published:** 2015-04-14

**Authors:** Ben S. Cooper, Maciej F. Boni, Wirichada Pan-ngum, Nicholas P. J. Day, Peter W. Horby, Piero Olliaro, Trudie Lang, Nicholas J. White, Lisa J. White, John Whitehead

**Affiliations:** 1 Mahidol Oxford Tropical Medicine Research Unit (MORU), Bangkok, Thailand; 2 Centre for Tropical Medicine and Global Health, Nuffield Department of Clinical Medicine, University of Oxford, Oxford, United Kingdom; 3 Oxford University Clinical Research Unit, Wellcome Trust Major Overseas Programme, Ho Chi Minh City, Viet Nam; 4 Department of Tropical Hygiene, Faculty of Tropical Medicine, Mahidol University, Bangkok, Thailand; 5 UNICEF-UNDP-World Bank-WHO Special Programme for Research and Training in Tropical Diseases, Geneva, Switzerland; 6 Department of Mathematics and Statistics, Lancaster University, Lancaster, United Kingdom; Stanford University, UNITED STATES

## Abstract

**Background:**

Experimental treatments for Ebola virus disease (EVD) might reduce EVD mortality. There is uncertainty about the ability of different clinical trial designs to identify effective treatments, and about the feasibility of implementing individually randomised controlled trials during an Ebola epidemic.

**Methods and Findings:**

A treatment evaluation programme for use in EVD was devised using a multi-stage approach (MSA) with two or three stages, including both non-randomised and randomised elements. The probabilities of rightly or wrongly recommending the experimental treatment, the required sample size, and the consequences for epidemic outcomes over 100 d under two epidemic scenarios were compared for the MSA, a sequential randomised controlled trial (SRCT) with up to 20 interim analyses, and, as a reference case, a conventional randomised controlled trial (RCT) without interim analyses.

Assuming 50% 14-d survival in the population treated with the current standard of supportive care, all designs had similar probabilities of identifying effective treatments correctly, while the MSA was less likely to recommend treatments that were ineffective. The MSA led to a smaller number of cases receiving ineffective treatments and faster roll-out of highly effective treatments. For less effective treatments, the MSA had a high probability of including an RCT component, leading to a somewhat longer time to roll-out or rejection. Assuming 100 new EVD cases per day, the MSA led to between 6% and 15% greater reductions in epidemic mortality over the first 100 d for highly effective treatments compared to the SRCT. Both the MSA and SRCT led to substantially fewer deaths than a conventional RCT if the tested interventions were either highly effective or harmful. In the proposed MSA, the major threat to the validity of the results of the non-randomised components is that referral patterns, standard of care, or the virus itself may change during the study period in ways that affect mortality. Adverse events are also harder to quantify without a concurrent control group.

**Conclusions:**

The MSA discards ineffective treatments quickly, while reliably providing evidence concerning effective treatments. The MSA is appropriate for the clinical evaluation of EVD treatments.

## Abstract 2

Ben Cooper and colleagues model different trial designs, including a multi-stage approach that contains non-randomized and randomized elements.

## Editors' Summary

**Background:**

The current outbreak of Ebola virus disease (EVD)—a frequently fatal disease that first appeared in human populations in 1976 in remote villages in central Africa—has infected more than 24,000 people and killed more than 10,000 people in Guinea, Sierra Leone, and Liberia since early 2014. Ebola virus is transmitted to people from wild animals and spreads in human populations through direct contact with the bodily fluids (including blood, saliva, and urine) or with the organs of infected people or through contact with bedding and other materials contaminated with bodily fluids. The symptoms of EVD which start 2–21 days after infection, include fever, headache, vomiting, diarrhea, and internal and external bleeding. Infected individuals are not infectious until they develop symptoms but remain infectious as long as their bodily fluids contain virus. There is no proven treatment or vaccine for EVD, but supportive care—given under strict isolation conditions to prevent the spread of the disease to other patients or to healthcare workers—improves survival.

**Why Was This Study Done?:**

Potential treatments for EVD include several antiviral drugs and injections of antibodies against Ebola virus from patients who have survived EVD. Before such therapies can be used clinically, their safety and effectiveness need to be evaluated, but experts disagree about how to undertake this evaluation. Drugs for clinical use are usually evaluated by undertaking a series of clinical trials. A phase I trial establishes the safety of the treatment and how the human body copes with it by giving several healthy volunteers the drug. Next, a phase II trial provides early indications of the drug’s efficacy by giving a few patients the drug. Finally, a large-scale multi-arm phase III randomized controlled trial (RCT) confirms the drug’s efficacy by comparing outcomes in patients randomly chosen to receive the investigational drug or standard care. This evaluation process is very lengthy. Moreover, it is hard to ethically justify undertaking an RCT in which only some patients receive a potentially life-saving drug during an Ebola epidemic. Here, the researchers evaluate a multi-stage approach (MSA) to EVD drug evaluation that comprises a single-arm phase II study followed by one or two phase III trials, one of which may be a sequential RCT (SRCT), a type of RCT that allows for multiple interim analyses, each of which may lead to study termination.

**What Did the Researchers Do and Find?:**

The researchers used analytic methods and computer simulations to compare EVD drug evaluation using the MSA, an SRCT, and a conventional RCT without interim analyses. Specifically, they estimated the probabilities of rightly or wrongly recommending the experimental treatment and the consequences for epidemic outcomes over 100 days for the three approaches. Assuming 50% survival at 14 days after symptom development in patients treated with supportive care only, all three trial designs were equally likely to identify effective treatments, but the MSA was less likely than the other designs to incorrectly recommend an ineffective treatment. Notably, the MSA led to fewer patients receiving ineffective treatments and faster roll-out of highly effective treatments. In an epidemic where 100 new cases occurred per day, for highly effective treatments, the MSA led to between 6% and 15% larger reductions in epidemic mortality over the first 100 days of the epidemic than the SRCT did. Finally, both the MSA and the SRCT led to fewer deaths than the conventional RCT if the tested interventions were either highly effective or harmful.

**What Do These Findings Mean?:**

These findings suggest that for experimental treatments that offer either no clinically significant benefit or large reductions in mortality the MSA can provide useful information about drug effectiveness faster than the other approaches tested. Thus, the MSA has the potential to reduce patient harm and the time to roll-out of an effective treatment for EVD. Although alternative evaluation designs are possible, the researchers suggest that including a non-randomized design in phase II is the quickest way to triage potential treatments and to decide how to test them further. For treatments that show strong evidence of benefit, it might even be possible to recommend the treatment without undertaking an RCT, they suggest. Moreover, for treatments that show only modest benefit in phase II, it should be easier (and more ethical) to set up RCTs to test the treatment further.

**Additional Information:**

Please access these websites via the online version of this summary at http://dx.doi.org/10.1371/journal.pmed.1001815.

- The World Health Organization (WHO) provides information about EVD, information about potential EVD therapies, and regular updates on the current EVD epidemic; a summary of the discussion of a WHO Ethics Working Group Meeting on the ethical issues related to study design for EVD drug trials is available; the WHO website also provides information about efforts to control Ebola in the field and personal stories from people who have survived EVD
- The UK National Health Service Choices website provides detailed information on EVD
- The US Centers for Disease Control and Prevention also provides information about EVD
- Wikipedia provides information about adaptive clinical trial design; a *Lancet* Global Health blog argues why adaptive trial designs should be used to evaluate drugs for the treatment of EVD

## Introduction

The largest ever outbreak of Ebola virus disease (EVD) is ongoing in west Africa, killing up to 70% of those infected [1,2]. Whilst there is no available vaccine and no proven treatments specific to EVD, there are several investigational treatments that might reduce mortality [3]. How should they be evaluated? Evaluations of novel treatments for EVD can take place only during an epidemic, and they need to have a high probability of identifying treatments able to provide clinically significant benefits, and a low probability of recommending ineffective or harmful interventions. They should produce results rapidly, to ensure maximum benefit (or minimum harm), and they need to be practical, implementable, and acceptable to those delivering and receiving care under very challenging conditions. Randomised controlled trials (RCTs) are the most reliable route to definitive answers on therapeutic benefits and harms, but there has been considerable debate about whether they can meet these additional needs in this EVD epidemic [4–6]. While some have argued that no other design would give reliable answers [5], others have countered that practical and ethical considerations mean that alternative study designs must also be considered [4]. In particular, when conventional care is associated with a very high probability of death, it may not be socially, operationally, or ethically acceptable to assign patients randomly to conventional care versus an experimental treatment that has a possibility of substantially increasing survival. Moreover, for investigational treatments that have a possibility of being highly effective (or highly harmful), using single-arm studies and adaptive designs (where enrolment depends on emerging efficacy data) as part of the evaluation process can reach conclusions faster, preventing unnecessary deaths.

In practice, drug development programmes seldom comprise a single clinical trial. Usually a series of studies is involved, with phase I establishing the safety and pharmacokinetic properties of the treatment and phase II providing early indications of efficacy, which, if found, are then confirmed in large-scale phase III trials. Typically, evidence from two phase III trials, or from a large and relevant phase II trial and one phase III trial, are required for a new drug to be licensed. In this paper we evaluate a multi-stage approach (MSA) to drug evaluation, where the first stage is a single-arm uncontrolled phase II study, which may lead on to the conduct of either one or two subsequent phase III trials, one of which may be a sequential RCT (SRCT). The performance of the MSA and potential impact on the current EVD epidemic is compared with the use of an SRCT alone or the use of a conventional RCT.

## Methods

Since most deaths from EVD occur within 14 d of admission to an Ebola treatment centre [1], all study designs we consider have survival to day 14 after randomisation (if performed) or admission to the treatment centre (otherwise) as the primary endpoint. Preliminary analysis of data from 1,820 adult patients with confirmed EVD and known disease outcome from four west African treatment centres shows that the proportion of patients surviving to 14 d lies consistently between 40% and 50% (personal communication Médecins Sans Frontières). Thus 50% is taken to be the 14-d survival for a new intervention to beat.

**Fig 1:**
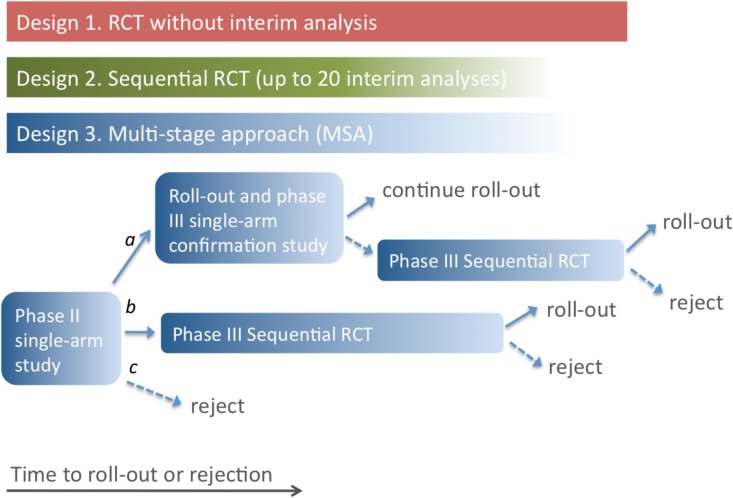
Three possible study designs. Design 1 uses an RCT with a predetermined number of participants, so time to roll-out or rejection of the treatment is determined only by the rate of enrolment of patients into the study. In Design 2, an interim analysis is performed for each additional group of 25 patient outcomes (group SRCT). For Design 3, if the single-arm phase II study indicates a large beneficial effect (conclusion a), the treatment is rolled out, and a phase III single-arm confirmation study is performed. Roll-out continues if this confirmation study gives a positive result; otherwise, an SRCT is employed. In the event of evidence of a moderate effect from the phase II study (conclusion b), the SRCT is employed. The treatment is rejected in the event of no evidence of benefit in the phase II study or negative results from an SRCT. In Designs 2 and 3, the number of participants recruited depends on the outcomes for patients already enrolled, and the study duration is uncertain (as indicated by the colour gradient).

Three possible study designs were compared for evaluating novel EVD treatments where the primary outcome was 14-d post-enrolment survival (Fig 1). Design 1 is a conventional RCT with no interim analysis, powered to give a 90% chance of detecting an increase in 14-d survival from 50% to 66.7% with a two-sided 5% significance level. We include Design 1 here to establish a baseline performance against which other designs are to be compared. We do not consider it an option that should be considered seriously. In practice, no trial in EVD would be conducted without a series of interim analyses and a pre-defined stopping rule.

Design 2 is a group SRCT that is particularly well-suited to situations where investigators wish to identify effective treatments but do not need to distinguish between ineffective and harmful treatments. It is based on the triangular test [7,8], with up to 20 interim analyses occurring every time 25 new day-14 records are received (so that a maximum of 500 patients are enrolled). The trial terminates when it is clear either that the treatment is better than the control or that evidence of superiority will not be found. The design is asymmetric, with higher power to detect a beneficial effect than a comparable harmful effect. This is appropriate because it is less important to determine whether a treatment offering no benefit is ineffective or harmful; in either case it should be rejected. The trial benefits from this asymmetry by having an expected sample size that is considerably smaller than rival group sequential approaches, especially when the treatment is less effective than control. The design is powered to match that of the conventional RCT, and provides a 90% chance of detecting an increase in 14-d survival from 50% to 66.7% with a two-sided 5% significance level.

Design 3, the MSA, uses an initial phase II trial where all patients receive the treatment. The trial is monitored continuously, and each time a new 14-d outcome is received, a decision is made (based on all 14-d outcomes received) to do one of the following: stop and conclude that the treatment is very effective (conclusion a), stop and conclude that the treatment is promising (conclusion b), stop and conclude that the treatment is ineffective (conclusion c), or continue to enrol patients. The stopping rules ensure that conclusion a is reached with probability 0.900 if treated patients have a 14-d survival rate of 80.0%, conclusion b is reached with probability 0.950 if the survival rate is 66.7%, and conclusion c is reached with probability 0.900 if the survival rate is 50.0%. Note that the MSA does not assume knowledge of the true standard care survival rate: if no RCT is performed, this rate is not estimated. The method is similar to the sequential medical plans of Bross [9] and is related to the double triangular test [10].

In the event of conclusion a, the treatment is rolled out immediately and further evaluated through a phase III single-arm confirmatory study using a form of futility design [11]. This phase III study is also monitored continuously, with a stopping rule that ensures that the treatment is confirmed as very effective with probability 0.900 if the 14-d survival rate is 80%. If this phase III study fails to confirm the treatment’s benefit, an SRCT is performed.

If conclusion b is reached in the phase II trial, the study proceeds directly to the phase III SRCT (Design 2). If conclusion c is reached, i.e., the SRCT or phase II single-arm trial provides no evidence of benefit, the treatment is rejected.

Full study details of all designs and stopping rules are provided in the section Study Protocol Details below.

For each of these three designs, for assumed survival rates in the treated population of between 10% and 90%, the following were determined: the probability of concluding that the treatment is effective, the sample size needed, the time to roll-out (if the treatment is not rejected), and the time to the final decision (assuming a capacity to enrol five patients in the study per day, based on assessment of potential study sites). The probability of concluding that the treatment is effective and the total sample size were computed for the different designs using methods that were either exact or provided very close approximations. Other quantities were determined by simulation. The potential impact of the three designs on mortality in the wider community amongst those with EVD onset in the 100 d following recruitment of the first patient was determined under two scenarios: one scenario assumed 100 new cases per day, and a more pessimistic scenario assumed an initial 200 cases per day, with a constant doubling period of 30 d. Treatment was assumed to be made available immediately to all new EVD cases after the roll-out time. Simulation results are based on 100,000 simulation runs for each value of the survival rate in the treated population. Simulations were performed using R [12]. Details are given in S1 Text.

### Study Protocol Details

For every patient, death or survival is recorded 14 d after entry into the trial. Even if deaths are known about earlier, they are not counted in the primary analysis until day 14. This is because we cannot assess survival until day 14.

Design 1 is a conventional RCT with 1:1 randomisation and no interim analysis. When 14-d outcomes have been obtained from all patients (*n* = 360), analysis is performed using Pearson’s chi-squared test without continuity correction.

**Fig 2:**
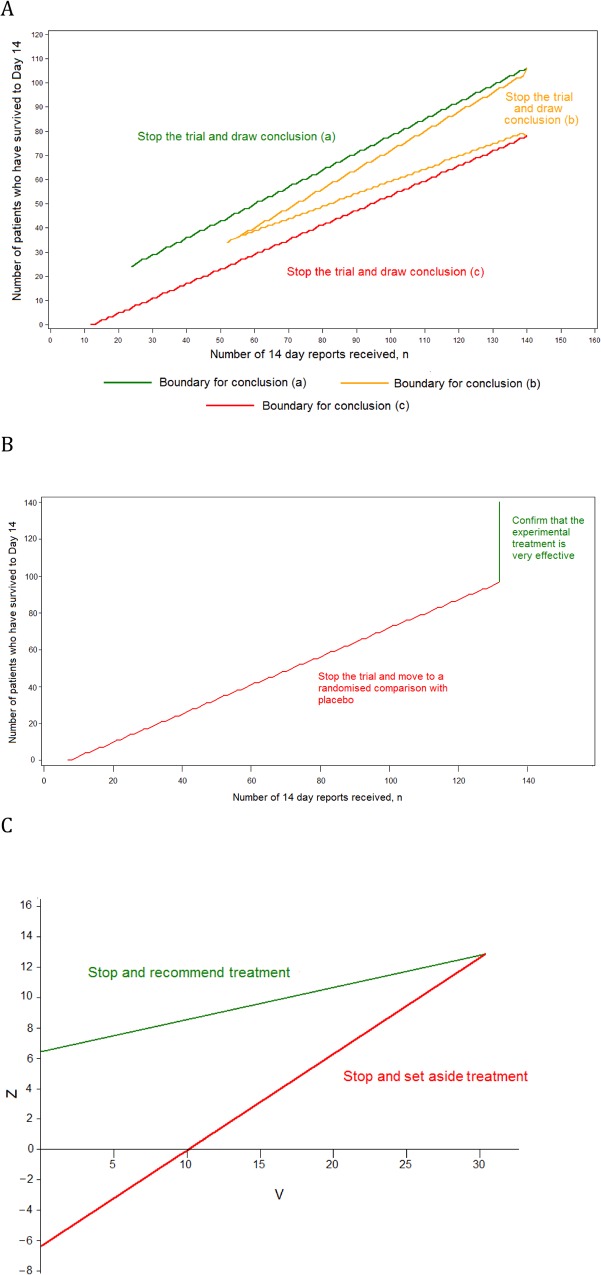
Stopping rules for the component trials of the multi-stage approach. (A) The single-arm phase II design, (B) the phase III single-arm confirmatory design, and (C) the phase III SRCT.

Design 2, a group SRCT, is also used as part of Design 3 (Fig 2). In Design 2, up to 20 interim analyses of the data are conducted. At each interim analysis (performed every time that 25 new day-14 records are received), the statistics *Z* and *V* are calculated. These are of the form given in Chapter 3.2 of Whitehead [7]. If sample sizes in the experimental treatment and control groups are equal, *Z* is simply the number of successes in the experimental treatment group minus the number in the control group, divided by two. The value of *V* is proportional to the sample size. The trial is stopped with the conclusion that the experimental treatment is significantly better than control if *Z* ≥ 6.3990 + 0.2105*V*. The trial is stopped with the conclusion that the experimental treatment is not significantly better than control if *Z* ≤ −6.3990 + 0.6315*V*. Otherwise, the trial is continued until the next interim analysis. These stopping rules are illustrated in Fig 2C. The design is set to conclude wrongly that the experimental treatment is better than control with probability 0.025 if the experimental treatment is actually associated with the same 14-d survival rate as the control, and to conclude rightly that the experimental treatment is better than the control with probability 0.900 if the odds ratio for 14-d survival is two. Such an advantage occurs if the 14-d survival rates for the experimental treatment and control are 66.7% and 50.0%, respectively, or if they are 80.0% and 66.7%. The final total sample size when using this design is likely to be between 100 and 200. The method is based on Whitehead [7,8].

Design 3, the MSA, has three component trials. The phase III SRCT is described above (Design 2). The phase II single-arm trial and the phase III single-arm confirmatory trial are described below.

For the phase II single-arm trial, all patients receive the experimental treatment, and a continuous record is maintained of the number of patients who have survived to day 14 (*S*) and the number of reports of day-14 status received (*n*). The record is used to determine when the trial should stop and what the conclusion should be, as follows:

- Conclusion a: if *n* ≥ 24 and *S* ≥ 7.117 + 0.7034*n*, then the trial stops with the conclusion that the experimental treatment is very effective. This is likely to occur if around 80% of patients survive to day 14.
- Conclusion b: if *n* ≥ 52 and either *S* ≤ −7.117 + 0.7970*n* or *S* ≥ 7.117 + 0.5164*n*, then the trial stops with the conclusion that the experimental treatment is promising. This is likely to occur if around 67% of patients survive to day 14.
- Conclusion c: if *n* ≥ 12 and *S* ≤ −7.117 + 0.6099*n*, then the trial stops with the conclusion that the treatment does not appear to be promising. This is likely to occur if around 50% of patients survive to day 14.

These stopping rules are shown graphically in Fig 2A. This design was chosen as it ensures that (1) conclusion a is reached with probability 0.900 if the 14-d survival rate is truly equal to 80.0%, (2) conclusion b is reached with probability 0.950 if the rate is 66.7%, and (3) conclusion c is reached with probability 0.900 if the rate is 50.0%. The maximum sample size is 140 patients, and the trial is most likely to stop after between 50 and 100 day-14 records have been received.

The phase III single-arm confirmatory trial recruits a maximum of 132 patients, all receiving the experimental treatment. This trial is also monitored continuously, and stops to reject the experimental treatment early if it is ever observed that *S* ≤ −5.2425 + 0.7747*n* (Fig 2B). The criteria chosen ensure that the experimental treatment is correctly confirmed as very effective with a probability 0.900 if the 14-d survival rate is 80.0% and is wrongly confirmed as very effective with probability 0.025 if the rate is 66.7%. The method is based on the futility design of Whitehead and Matsushita [11].

The final analysis of the group SRCT that forms Design 2 and features as the third component of Design 3 is designed to avoid potential biases due to the use of the stopping rule [7,13]. The final analysis of the phase II single-arm component of Design 3 uses the stopping rule employed by Jovic and Whitehead [14], which is a generalisation of the better known exact approach of Clopper and Pearson [15].

### Ethics Statement

An ethics statement was not required for this work.

## Results

**Fig 3:**
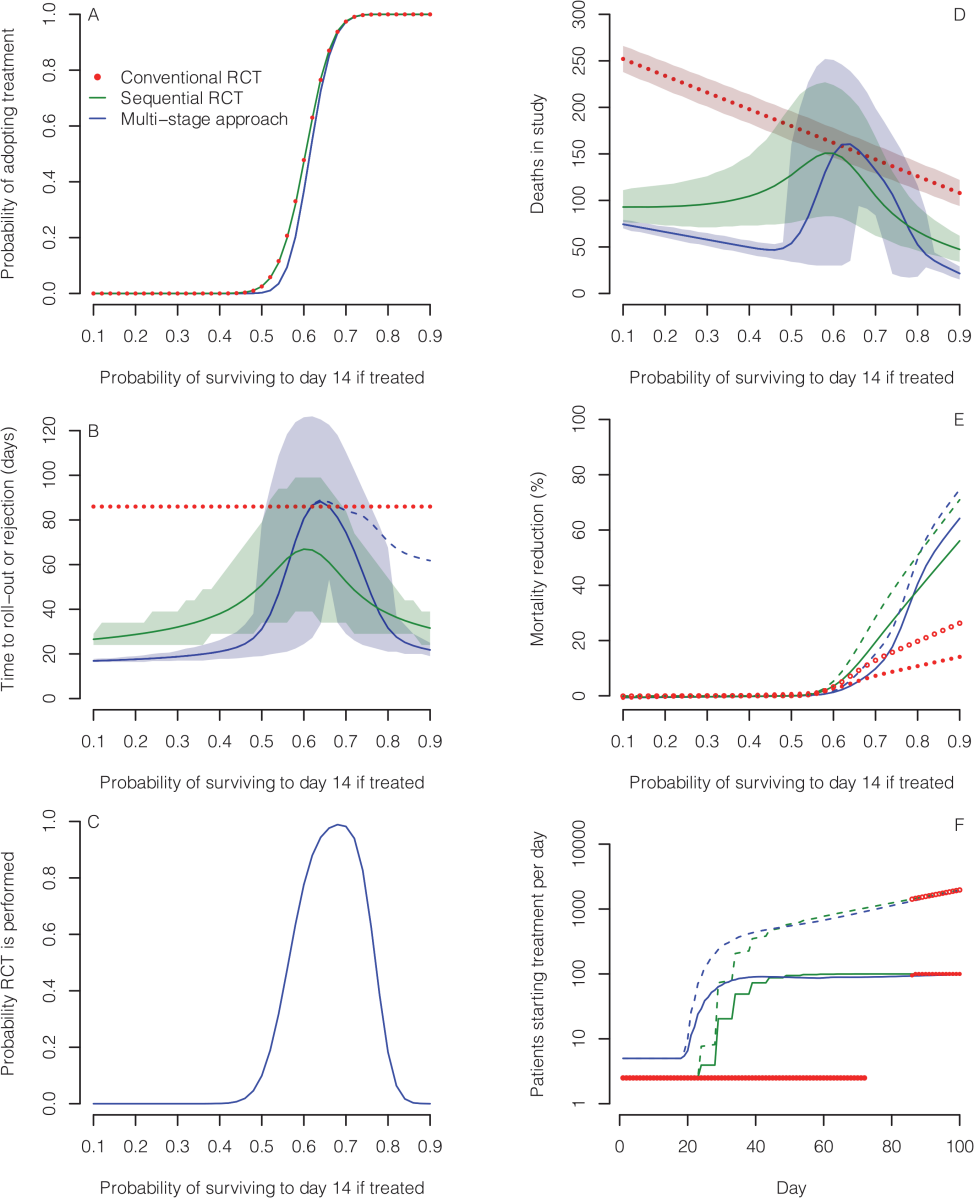
Comparison of the three designs for evaluating treatments assuming 50% 14-d survival probability in the standard care group. The designs compared are a conventional RCT without interim analysis (red), a group SRCT with interim analyses for every 25 patients with 14-d outcomes (green), and an MSA (blue). (A) Probability of concluding an experimental treatment is effective (defined as a treatment where the probability of surviving to day 14 is greater than 0.5). (B) Mean time to rolling out or discarding the treatment (solid and dotted lines) and associated 5th and 95th percentiles (shaded regions). In the MSA, a phase III single-arm confirmation study may be performed following roll-out. Time to reach a conclusion from this study is shown by the dashed line. (C) Probability that an RCT is performed in the MSA. (D) Mean number of deaths amongst patients enrolled for the three study designs. (E) Mean percentage reduction in mortality amongst EVD cases in the wider population over 100 d following the start of evaluation compared with no treatment scenarios. Results are shown for two scenarios: one scenario assumes 100 new cases per day (solid lines and filled circles), and the other assumes exponential epidemic growth (dashed lines and open circles). Treatment is assumed to be made available immediately to all new EVD cases after the roll-out time. (F) Mean number of patients starting treatment each day under the scenarios in (E) assuming that the experimental treatment increases 14-d survival probability to 80%.

Based on the assumption that a patient with EVD in west Africa has a 50% chance of surviving to day 14 with standard care alone, all three designs gave similar probabilities of identifying effective treatments correctly, and low probabilities of recommending those that were ineffective or harmful. The MSA was least likely to lead to the recommendation of ineffective treatments or of those conferring only a small benefit (Table 1; Fig 3A). This was also true if standard care day-14 survival was only 40% (S1 Fig).

**Table 1:** Probabilities of recommending treatment and mean sample size for the three different study designs for various combinations of survival rates.

Probability of Surviving to Day 14		Design 1: Conventional RCT		Design 2: SRCT		Design 3: MSA	
Control	Experimental Treatment	Probability of Recommending Experimental Treatment	Sample Size	Probability of Recommending Experimental Treatment	Mean Sample Size	Probability of Recommending Experimental Treatment	Mean Sample Size
0.500	0.500	0.026	360	0.025	182	0.002	79
0.500	0.667	0.897	360	0.900	225	0.885	292
0.500	0.800	1.000	360	1.000	115	1.000	204
0.667	0.500	0.000	360	0.000	96	0.000	70
0.667	0.667	0.026	360	0.025	205	0.025	273
0.667	0.800	0.819	360	0.900	279	0.982	234
0.667	0.889	0.999	360	1.000	151	1.000	171

If the true survival rate without the experimental treatment was better than anticipated (66.7% instead of 50%), then the probability of wrongly recommending an ineffective treatment was 0.025 in all three designs, while MSA had substantially greater power to recommend a treatment that increased survival to about 80% (Table 1; S2 Fig). The expected sample sizes using the MSA (including the patients recruited for the phase III single-arm confirmatory trial, if used) were considerably smaller than those required for the single-stage designs if the treatment was ineffective, although they could be larger in the case of effective treatments. However, sample sizes and times to reach a conclusion varied substantially (Fig 3B). A conventional RCT would require about 12 wk to recruit and follow up the required 360 participants. For the other two designs, expected study duration depended on treatment effectiveness, though in all cases the mean duration was substantially shorter than with the conventional RCT. For very effective or ineffective treatments, the MSA gave the most rapid time to roll-out or rejection, respectively (Fig 3B). For promising treatments, the phase II study in the MSA usually led to conclusion b, leading to an SRCT (Fig 3C). This resulted in longer times to reach a conclusion, and under these circumstances the SRCT typically had the shortest time to roll-out or rejection. Estimated numbers of deaths amongst those enrolled into the study were highest for the conventional RCT and lowest for the MSA for ineffective and very effective treatments (Fig 3D). For promising treatments, they were similar in the SRCT and the MSA.

The more rapidly an effective treatment can be rolled out to patients outside a study population, the greater the overall mortality reduction in an epidemic. As a consequence, because it takes longer to reach a conclusion, the conventional RCT compares poorly with the other designs for reducing total mortality (Fig 3E). For very effective treatments (where the probability of survival if treated is at least 80%), the potential for mortality reduction is substantial with the other two designs under both epidemic scenarios (Fig 3E). Under the scenario of 100 new cases per day, using the MSA led to a reduction in mortality for very effective treatments that was between 6% and 15% higher than that achieved by the SRCT; for a treatment conferring an 80% or 90% 14-d survival probability and 100% treatment take-up, this corresponds to an average of 124 and 434 additional deaths prevented over 100 d, respectively. The corresponding deaths prevented by using the MSA instead of a conventional RCT were 1,589 and 2,673. For less effective treatments, a greater mortality reduction could be achieved using the SRCT, owing to its faster rollout. For example, for a treatment conferring a 70% survival probability, compared to a 50% survival probability without the treatment, the mortality reduction achieved over 100 d was 10% with the MSA but 19% with the SRCT, corresponding to 515 fewer deaths. If a treatment is effective, then for the potential benefits of the MSA and SRCT to be realised, large numbers of treatment courses need to be made available early on (Fig 3F).

The evaluation of multiple experimental treatments in series or parallel was not considered in detail in this study, but suppose that treatments A, B, and D are tested one after the other (where treatment C is the control). Suppose further that the 14-d survival rates for treatments A, B, and D are 50.0%, 50.0%, and 80.0%, respectively, while the survival rate for the control is 50.0%. The MSA correctly recommends only treatment D with probability 0.996, after an expected sample size of 361. The SRCT recommends only treatment D with probability 0.951, after an expected sample size of 479.

## Discussion

While the three designs have similar power to detect effective EVD treatments if day-14 survival in the standard care population is 50%, their performances differ importantly in other respects. In particular, adaptive designs are likely to produce results faster than a conventional RCT, and if the treatment is ineffective or very effective, the MSA is likely to give the shortest time to rejection or roll-out, respectively. If day-14 survival in the standard care population is 66.7%, the MSA has the same probability of wrongly recommending ineffective treatments as the other two designs, but can have substantially greater power to detect effective treatments. For promising treatments (e.g., those that increase day-14 survival from 50.0% to 66.7%), the MSA has a high probability of including a randomised trial, though this comes at the cost of somewhat longer times to reach a conclusion than the SRCT.

Our analysis assumed equal rates of recruitment in all study designs. In practice, difficulties in recruiting patients into randomised trials might give the MSA a further advantage. For harmful treatments, the MSA minimises in-study deaths and wasted investigator effort. If a number of treatments are to be evaluated in sequence, the MSA could reduce substantially the time needed to find one that is effective. For beneficial treatments, the MSA allows faster roll-out to the wider population.

RCTs are usually the best method for evaluating interventions because, if conducted well, they minimise the potential for reaching incorrect conclusions by controlling for both known and unknown confounders. However, the current Ebola epidemic in west Africa is an unprecedented situation where there is substantial uncertainty that RCTs can be conducted successfully and safely [3]. RCTs have been conducted for diseases with high fatality (e.g., trauma), but never before in a setting where there are major safety concerns for healthcare workers (where every procedure involves a life-threatening risk), additional ethical dilemmas (such as the likelihood of having to randomise family members), and widespread mistrust amongst the community. Given these operational concerns and the results of our analysis, the MSA—which begins with a less operationally challenging design and yet retains the ability to provide robust and informative results—must be considered. Although the single-arm phases of the MSA are more vulnerable to spurious conclusions if the case fatality rate varies for reasons unrelated to the intervention, the risk of an incorrect conclusion depends on the true effect size. For larger effect sizes (whether harmful or beneficial), the risk is smaller. Indeed, decisions to discard or pursue interventions further with evidence of large harmful effects or large beneficial effects are often made without evidence from RCTs (e.g., in early evaluations of new drugs for treatment of cancers with poor prognosis).

In the proposed MSA, the major threats to the validity of the results of the non-randomised components are that referral patterns, standard of care, or the virus itself may change during the study period in ways that affect mortality. However, the stopping rules applied in this analysis are likely to be robust to fluctuations in case fatality rate, since a 50% survival rate has been achieved with the current standard of care in west Africa, whereas an 80% survival rate has not been observed to our knowledge in any west African Ebola treatment centre. Our results also show that even if the baseline 14-d survival rate increases by more than 30% (to 66.7%), the MSA has only a 2.5% chance of recommending an ineffective treatment and a much greater chance of correctly recommending a treatment that increased the survival rate to 80%. An acknowledged weakness of the uncontrolled design is that adverse events are much harder to quantify without a concurrent control group. However, in the context of an illness with a fatality risk of 50% or greater and very limited possibilities to monitor adverse events, life-threatening adverse events are the most relevant safety measure, and these are captured with the fatality endpoint.

Many alternative designs are, of course, possible. For example, response adaptive designs aim to reduce the number of patients receiving ineffective treatment by putting more patients in the better treatment arm based on results accrued in the trial. However, such designs provide little further benefit when used in conjunction with sequential procedures. Moreover, when there is a substantial delay from treatment allocation to the primary response (as in our case), the scope for using emerging data in determining the treatment allocation of subsequent patients will be limited [16,17].

The MSA might lead to the identification of more than one promising treatment, or other trial groups might find such treatments. Then the SRCT component in the MSA could be replaced by a clinical comparison of more than one active treatment using a multi-arm multi-stage (MAMS) design [18]. Unless any of the new treatments have been found to be very effective (conclusion a in our scheme), a standard therapy arm should also be included.

Finally, it is conceivable that an intervention may improve 14-d mortality but worsen overall mortality. Whatever the study design, it will therefore be important to monitor 30-d mortality as a secondary outcome measure.

In summary, our results show that in the case of experimental treatments offering either no clinically significant benefit or large reductions in mortality, the MSA can reach a conclusion faster than the other approaches, minimising patient harm, wasted investigator effort, or time to roll-out. While some have objected to the use of evidence from studies where patients have not been randomised to interventions, we strongly believe the unrandomised design in phase II (as we propose) is appropriate. It is the quickest way to triage the treatments and decide how to test them further. The MSA does have the capacity to proceed to recommend an experimental treatment without the conduct of an RCT. However, this will happen only following strong and confirmed evidence of a success rate that is well in excess of that which has been observed with conventional treatment. For treatments with more modest benefits, an RCT will be needed as the second stage of the procedure. It is hoped that, having ruled out exceptional benefit, the barriers to randomisation might then be overcome more easily.

## Supporting Information

S1 Fig Comparison of the three designs for evaluating treatments assuming a 40% 14-d survival probability in the standard care group. (TIF)

S2 Fig Comparison of the three designs for evaluating treatments assuming a 66.7% 14-d survival probability in the standard care group. (TIF)

S1 Text Simulation details. (DOCX)

## Abbreviations:

EVD: Ebola virus disease
MSA: multi-stage approach
RCT: randomised controlled trial
SRCT: sequential randomised controlled trial

## References

[1] WHO Ebola Response Team (2014) Ebola virus disease in West Africa—the first 9 months of the epidemic and forward projections. N Engl J Med 371: 1481–1495. 10.1056/NEJMoa1411100 25244186 PMC4235004

[2] Farrar JJ, Piot P (2014) The Ebola emergency—immediate action, ongoing strategy. N Engl J Med 371: 1545–1546. 10.1056/NEJMe1411471 25244185

[3] Heymann DL (2014) Ebola: learn from the past. Nature 514: 299–300. 10.1038/514299a 25318509

[4] Adebamowo C, Bah-Sow O, Binka F, Bruzzone R, Caplan A, et al (2014) Randomised controlled trials for Ebola: practical and ethical issues. Lancet 384: 1423–1424. 10.1016/S0140-6736(14)61734-7 25390318 PMC4392883

[5] Joffe S (2014) Evaluating novel therapies during the Ebola epidemic. JAMA 312: 1299–1300. 10.1001/jama.2014.12867 25211645

[6] Rid A, Emanuel EJ (2014) Ethical considerations of experimental interventions in the Ebola outbreak. Lancet 384: 1896–1899. 10.1016/S0140-6736(14)61315-5 25155413

[7] Whitehead J (1997) The design and analysis of sequential clinical trials, revised 2nd edition Chichester: Wiley.

[8] Whitehead J (2011) Group sequential trials revisited: simple implementation using SAS. Stat Methods Med Res 20: 635–656. 10.1177/0962280210379036 20876163

[9] Bross I (1952) Sequential medical plans. Biometrics 8: 188–205.

[10] Whitehead J, Todd S (2004) The double triangular test in practice. Pharm Stat 3: 39–49.

[11] Whitehead J, Matsushita T (2003) Stopping clinical trials because of treatment ineffectiveness: a comparison of a futility design with a method of stochastic curtailment. Stat Med 22: 677–687. 12587099 10.1002/sim.1429

[12] R Core Team (2014) R: a language and environment for statistical computing Vienna: R Foundation for Statistical Computing.

[13] Fairbanks K, Madsen R (1982) P values for tests using a repeated significance test design. Biometrika 69: 69–74.

[14] Jovic G, Whitehead J (2010) An exact method for analysis following a two-stage phase II cancer clinical trial. Stat Med 29: 3118–3125. 10.1002/sim.3837 21170906

[15] Clopper CJ, Pearson ES (1934) The use of confidence or fiducial limits illustrated in the case of the binomial. Biometrika 26: 404–413.

[16] Rout CC, Rocke DA, Levin J, Gouws E, Reddy D (1993) A reevaluation of the role of crystalloid preload in the prevention of hypotension associated with spinal anesthesia for elective cesarean section. Anesthesiology 79: 262–269. 8192733 10.1097/00000542-199308000-00011

[17] Rosenberger WF, Stallard N, Ivanova A, Harper CN, Ricks ML (2001) Optimal adaptive designs for binary response trials. Biometrics 57: 909–913. 11550944 10.1111/j.0006-341x.2001.00909.x

[18] Magirr D, Jaki T, Whitehead J (2012) A generalized Dunnett test for multiarm-multistage clinical studies with treatment selection. Biometrika 99:494–501.

