# Supplementary figures and images for: Evaluating Clinical Trial Designs for Investigational Treatments of Ebola Virus Disease
Source: PLoS Med. 2015 Apr 14;12(4):e1001815. doi: 10.1371/journal.pmed.1001815 (PMC4397078; doi:10.1371/journal.pmed.1001815)

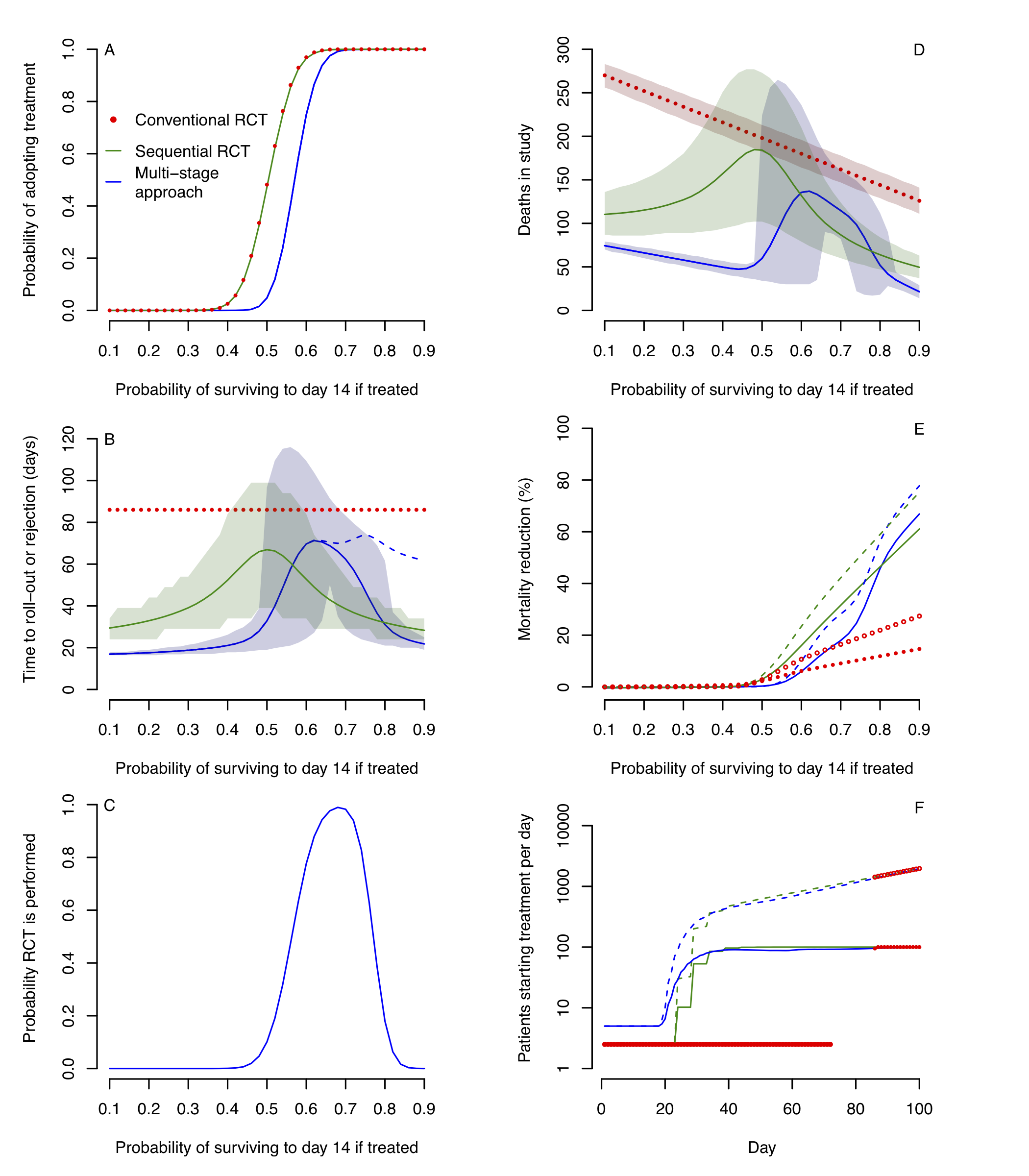

Supplement: S1 Fig — (TIF) [file pmed.1001815.s001.tif]

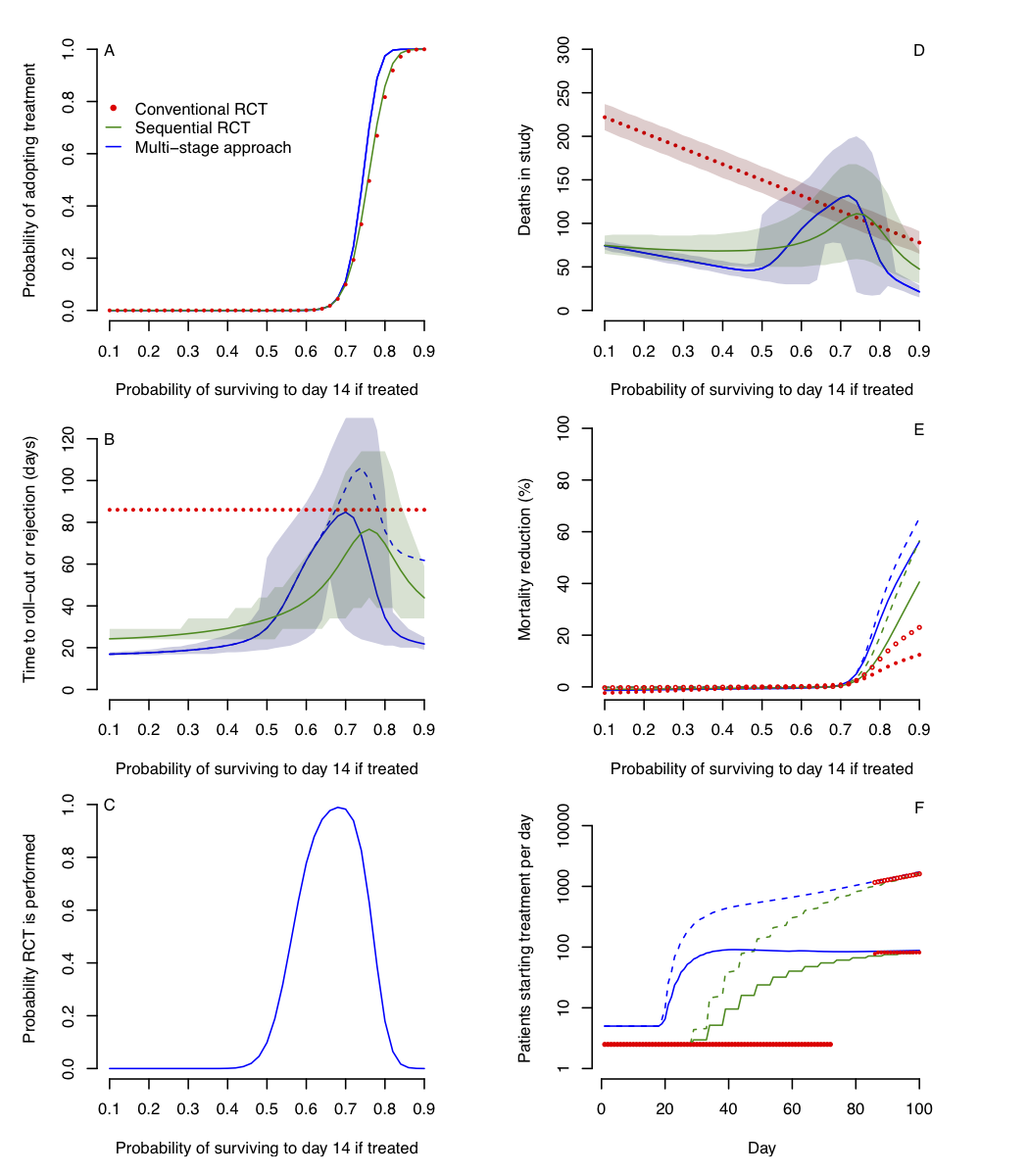

Supplement: S2 Fig — (TIF) [file pmed.1001815.s002.tif]
