# Supplementary material for: Evaluating Clinical Trial Designs for Investigational Treatments of Ebola Virus Disease
Source: PLoS Med. 2015 Apr 14;12(4):e1001815. doi: 10.1371/journal.pmed.1001815 (PMC4397078; doi:10.1371/journal.pmed.1001815)
Supplement: S1 Text — (DOCX) [file pmed.1001815.s003.docx]

**Simulation details for Figure 3, S1 and S2**

Simulations for Figures 3, S1 and S2 were performed using assumptions in Table S1 below. In the case of randomized designs, synthetic data were generated by randomly assigning patients to treatment or control (each with probability 0.5). Day 14 outcomes were randomly generated for all patients using appropriate probabilities for treated and standard-of-care patients. These synthetic data were then analysed using the approaches described in detail in Supplementary Protocol S1. Analysis was performed in R version 3.1.1 ( http://www.R-project.org/) . Code is available at <http://benscooper.github.io/Clinical-Trials-Designs-for-Ebola-Treatment/> .

**Table S1: Simulation details for Figure 2 of the paper**

|  | Value or range |
| --- | --- |
| Case fatality with standard care14 days post-enrolment | 40.0 to 66.7% |
| Proportion of total case fatality occurring by day 14 | 94% |
| Case fatality with treatment 14 days post-enrolment | 10-90% |
| Number of patients recruited per day | 5 |
| Number of EVD cases per day: scenario 1 | 100 |
| Number of EVD cases per day at start of study: scenario 2 | 200 |
| Epidemic doubling time under scenario 2 | 30 days |

**References**

R Core Team (2014). R: A language and environment for statistical computing. R Foundation for Statistical Computing, Vienna, Austria. URL
